# Crestal and Subcrestal Placement of Morse Cone Implant–Abutment Connection Implants: An In Vitro Finite Element Analysis (FEA) Study

**DOI:** 10.3390/biomedicines11113077

**Published:** 2023-11-16

**Authors:** Luca Comuzzi, Mario Ceddia, Natalia Di Pietro, Francesco Inchingolo, Angelo Michele Inchingolo, Tea Romasco, Margherita Tumedei, Alessandro Specchiulli, Adriano Piattelli, Bartolomeo Trentadue

**Affiliations:** 1 Independent Researcher, San Vendemiano-Conegliano, 31020 Treviso, Italy; luca.comuzzi@gmail.com; 2 Department of Mechanics, Mathematics and Management, Polytechnic University of Bari, 70125 Bari, Italy; marioceddia1998@gmail.com (M.C.); bartolomeo.trentadue@poliba.it (B.T.); 3 Department of Medical, Oral and Biotechnological Sciences, “G. d’Annunzio” University of Chieti-Pescara, 66100 Chieti, Italy; tea.romasco@unich.it (T.R.); alessandrospecchiulli@alice.it (A.S.); 4 Center for Advanced Studies and Technology (CAST), “G. d’Annunzio” University of Chieti-Pescara, 66100 Chieti, Italy; 5 Department of Interdisciplinary Medicine, University of Bari “Aldo Moro”, 70121 Bari, Italy; francesco.inchingolo@uniba.it (F.I.); angeloinchingolo@gmail.com (A.M.I.); 6 Department of Medical, Surgical and Dental Sciences, University of Milan, 20122 Milan, Italy; margherita.tumedei@unimi.it; 7 School of Dentistry, Saint Camillus International University of Health and Medical Sciences, 00131 Rome, Italy; apiattelli51@gmail.com; 8 Facultad de Medicina, UCAM Universidad Católica San Antonio de Murcia, 30107 Murcia, Spain

**Keywords:** bone resorption, Morse cone connection, dental implants, finite element analysis (FEA), peri-crestal stress, subcrestal positioning

## Abstract

The issue of dental implant placement relative to the alveolar crest, whether in supracrestal, equicrestal, or subcrestal positions, remains highly controversial, leading to conflicting data in various studies. Three-dimensional (3D) Finite Element Analysis (FEA) can offer insights into the biomechanical aspects of dental implants and the surrounding bone. A 3D model of the jaw was generated using computed tomography (CT) scans, considering a cortical thickness of 1.5 mm. Subsequently, Morse cone implant–abutment connection implants were virtually positioned at the model’s center, at equicrestal (0 mm) and subcrestal levels (−1 mm and −2 mm). The findings indicated the highest stress within the cortical bone around the equicrestally placed implant, the lowest stress in the −2 mm subcrestally placed implant, and intermediate stresses in the −1 mm subcrestally placed implant. In terms of clinical relevance, this study suggested that subcrestal placement of a Morse cone implant–abutment connection (ranging between −1 and −2 mm) could be recommended to reduce peri-implant bone resorption and achieve longer-term implant success.

## 1. Introduction

Crestal bone resorption around dental implants is a common occurrence, often attributed to a multifactorial pattern involving factors such as overloading, colonization of the micro-gap at the implant–abutment junction, reformation of the biological width, or surgical trauma [1,2,3,4]. One of the primary contributions to marginal bone resorption is believed to be the unfavorable transmission of masticatory loads at the bone–implant interface [5].

According to the remodeling theory proposed by Frost in 1964 [6], bone reshapes itself on the implant by adapting to the applied loads. Frost distinguished between internal and external remodeling. Internal remodeling involves changes in density, while external remodeling results in changes in morphology when the bone is exposed to certain loading conditions. Within a stress range of 11–30 MPa, the bone is typically in a state of equilibrium (lazy zone), maintaining stable conditions, referred to as the homeostatic state. Exceeding this equilibrium may lead to bone deposition, while stress beyond a certain range can cause bone microfractures and subsequent resorption due to overload. Conversely, stress lower than the equilibrium value may result in decreased mineral density and bone atrophy. Various studies have been conducted to analytically describe bone remodeling as a function of load, highlighting the critical role of efficient load transmission from the implant to limit bone resorption [6]. Treatment plans should incorporate methods to reduce stress, minimizing the likelihood of initial bone loss. Biomechanical techniques, such as increasing implant surface area, can be employed to improve the condition of the transosseous region and lower crestal stress around implants. The type of implant placement also influences force transmission to the bone, with greater bone–implant contact leading to increased rigidity and altered stress [7].

Within implant dentistry, a contentious issue revolves around the placement of dental implants in relation to the alveolar crest, specifically in supracrestal, equicrestal, and subcrestal positions. In this context, the literature studies present conflicting results, leaving this matter without a definitive resolution [8,9]. Furthermore, it is crucial to consider that various implant–abutment connections are available, each resulting in distinct microbiological, clinical, and radiological outcomes. Studies on bacterial leakage have shown that both internal and external hexagonal connections exhibit high permeability to bacterial penetration [10]. When placing an implant with such a connection below the alveolar crest level, the micro-gap, susceptible to bacterial colonization, inevitably shifts in a more apical direction. This shift could potentially contribute to increased peri-crestal bone resorption [11,12,13,14]. In contrast, Morse cone or conical connection implant–abutment assemblies have demonstrated greater resistance to bacterial colonization [10]. The conometric connection mechanism is designed to establish a secure and durable connection between the implant and the denture. Notably, this connection allows for easy removal during maintenance or replacement of the prosthesis while still providing long-term stability and functionality. Inserting these implant–abutment junctions in a subcrestal position has been associated with lesser or no peri-implant bone resorption [15,16]. The necessity for a more apical placement of the micro-gap is primarily linked to achieving an improved and aesthetically pleasing prosthetic emergence profile, thereby reducing the risk of exposing the implant threads [15,17,18].

Finite Element Analysis (FEA) studies have proven to be highly valuable for assessing the biomechanical performance of dental implants in various experimental settings [19,20,21,22,23].

Thus, the primary objective of this study was to perform FEA on Morse cone connection implants, investigating their behavior when inserted equicrestally and subcrestally at depths of 1 and 2 mm below the crest level. The null hypothesis of this study posits that the placement of the implant in either a crestal or subcrestal manner does not influence the distribution of stress.

## 2. Materials and Methods

### 2.1. Modeling

**Figure 1:**
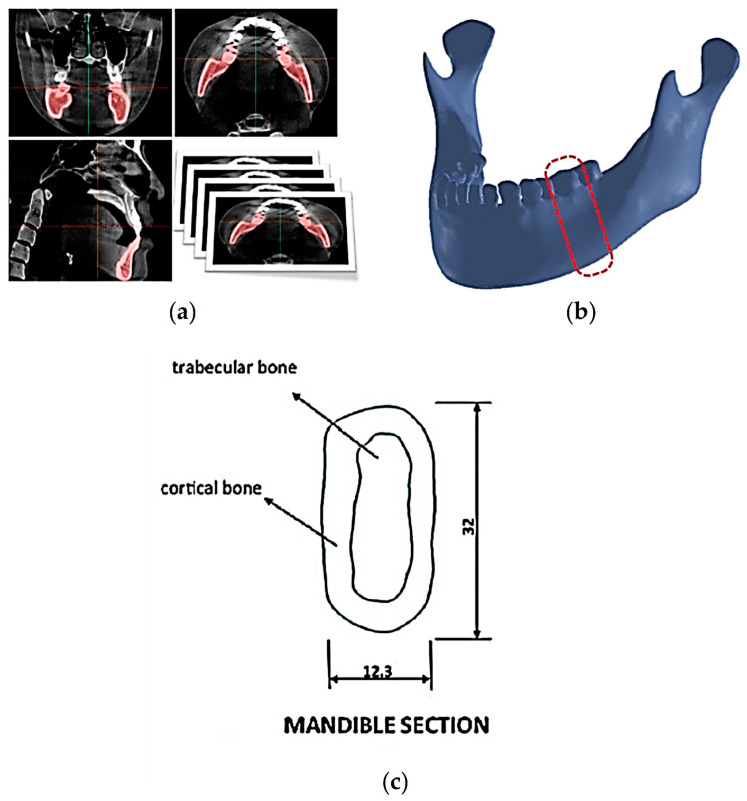
Modeling of a three-dimensional (3D) Finite Element for a single surgical model. (**a**) Extraction of geometry data from cone-beam computed tomography (CBCT) images. (**b**) Reconstruction of the surface and volume of the mandibular bone. The part circled in red has been considered for the study. (**c**) Selection of the bone section for the biomechanical study of implants. All dimensions are expressed in mm.

A three-dimensional (3D) model of the jaw was obtained through a cone-beam computed tomography (CBCT) scan (NewTom Giano HR, Cefla, Imola, Italy) [20,21], as represented in Figure 1.

This model was used to extract the accurate geometries of the analyzed bone, with particular attention to the employed cortical thickness of 1.5 mm for the biomechanical study of implants.

**Figure 2:**
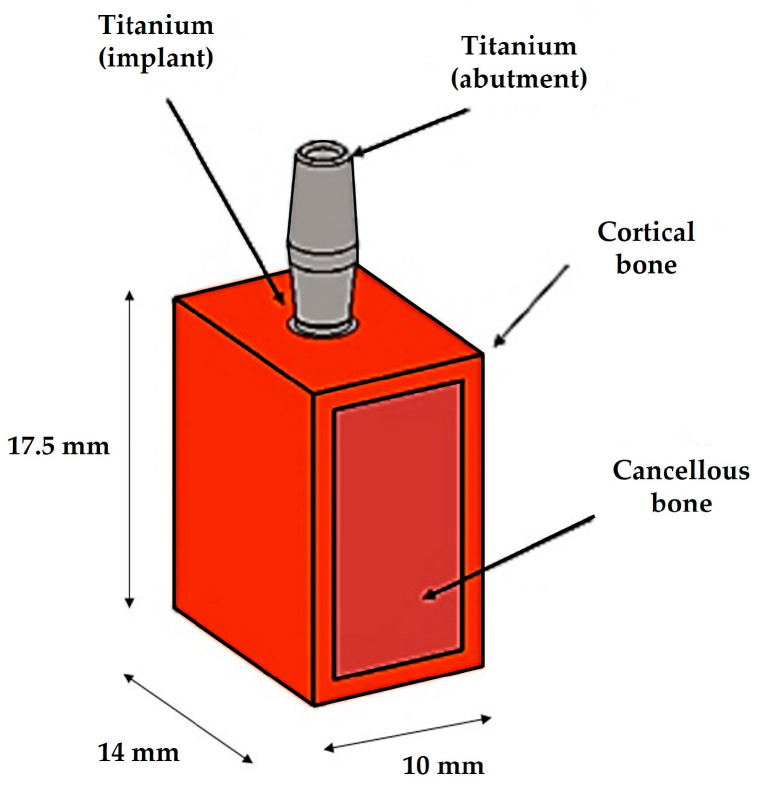
**A** simplified model comprising cortical bone, trabecular bone, and the inserted implant.

A computer-aided design (CAD) software (Autodesk Inventor 2023, San Francisco, CA, USA) was used to conduct the 3D modeling of the geometries corresponding to clinical cases reported in several studies [15,16,18]. For simplicity, a bone block model was constructed, measuring 17.5 mm in height, 10 mm in length, and 14 mm in width. The two bone models, representing cortical bone in orange and cancellous bone in pink, were paired using the Assemble command of the software, whereas implants were fixed in the bone using a full bounded mode to simulate complete osseointegration (Figure 2).

**Figure 3:**
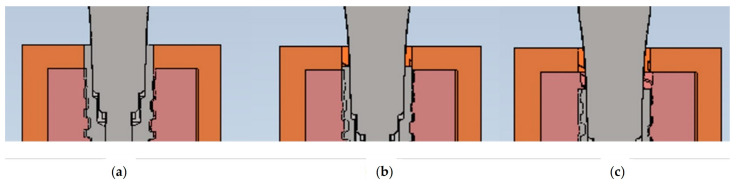
Equicrestal and subcrestal implant positioning: (**a**) 0 mm, (**b**) −1 mm, and (**c**) −2 mm. Cancellous bone is represented in pink, cortical bone in orange, and the implant and abutment in grey.

The investigated implants (AoN Implants Srl, Grisignano di Zocco, Italy) had a diameter of 3.5 mm and a length of 13 mm, besides presenting a Morse cone connection with a platform switch. Their models were virtually positioned at the center of this block at equicrestal (0 mm) and subcrestal levels (−1 mm and −2 mm) using the same CAD software (Autodesk Inventor 2023, San Francisco, CA, USA) (Figure 3).

### 2.2. Materials

For this study, a D2 bone type was considered according to the classification by Misch [4]. Properties of cortical bone, trabecular bone, and titanium implants were obtained from the literature and other biomechanical studies and assigned to the models [20,21,22,23]. Each solid component was modeled with isotropic, homogeneous, and linearly elastic behaviors to simplify the modeling. Therefore, Table 1 provides Young’s modulus (to measure stiffness) and Poisson’s ratio (to measure deformability) for each material.

### 2.3. Finite Element Analysis (FEA)

For the FEA of implant systems studied here, both equicrestal and subcrestal placements were simulated using Ansys Workbench 2023 software (Canonsburg, PA, USA). The mechanical properties of the structural materials (titanium and bone) were implemented in the software based on data obtained from Table 1.

The FEA method involves discretizing the continuous body into small elements called meshes and then applying elasticity equations to each element to determine the behavior of the body. The size of the mesh is crucial for obtaining accurate results [24,25,26]. In the present research, a linear tetrahedral mesh with a size of 0.5 mm was generated, as it enables more precise convergence on Von Mises stress, as elucidated by Gatti et al. [27]. The mesh was gradually refined at the implant/bone interface, with the element size reduced to 0.2 mm. This smallest element size is of the same order of dimension as the implant thread. Additionally, it has been observed that using a 0.2 mm mesh allows for good convergence in stress/strain results.

**Figure 4:**
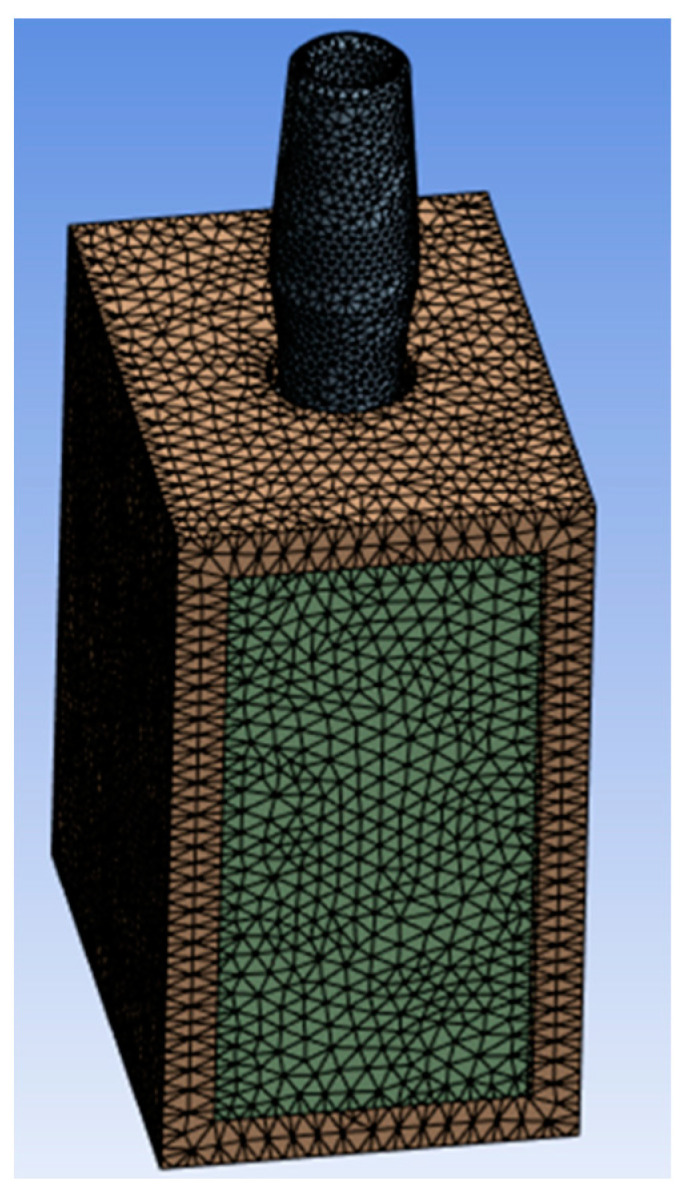
Finite Element Model (FEM) with a mesh size of 0.5 mm and 0.2 mm.

The geometric features and their representation are shown in Figure 4.

### 2.4. Loads and Constrains

The interface between the cortical bone and the trabecular bone, as well as between the implant and each layer of bone, was assumed to be fully constrained, corresponding to complete osseointegration. Thus, an ideal state of osseointegration was assumed at the bone–implant interface, where the bone completely enveloped the ridge of the implant thread. Therefore, concerning the contact aspect, fixation was applied in all three directions relative to the screw.

**Figure 5:**
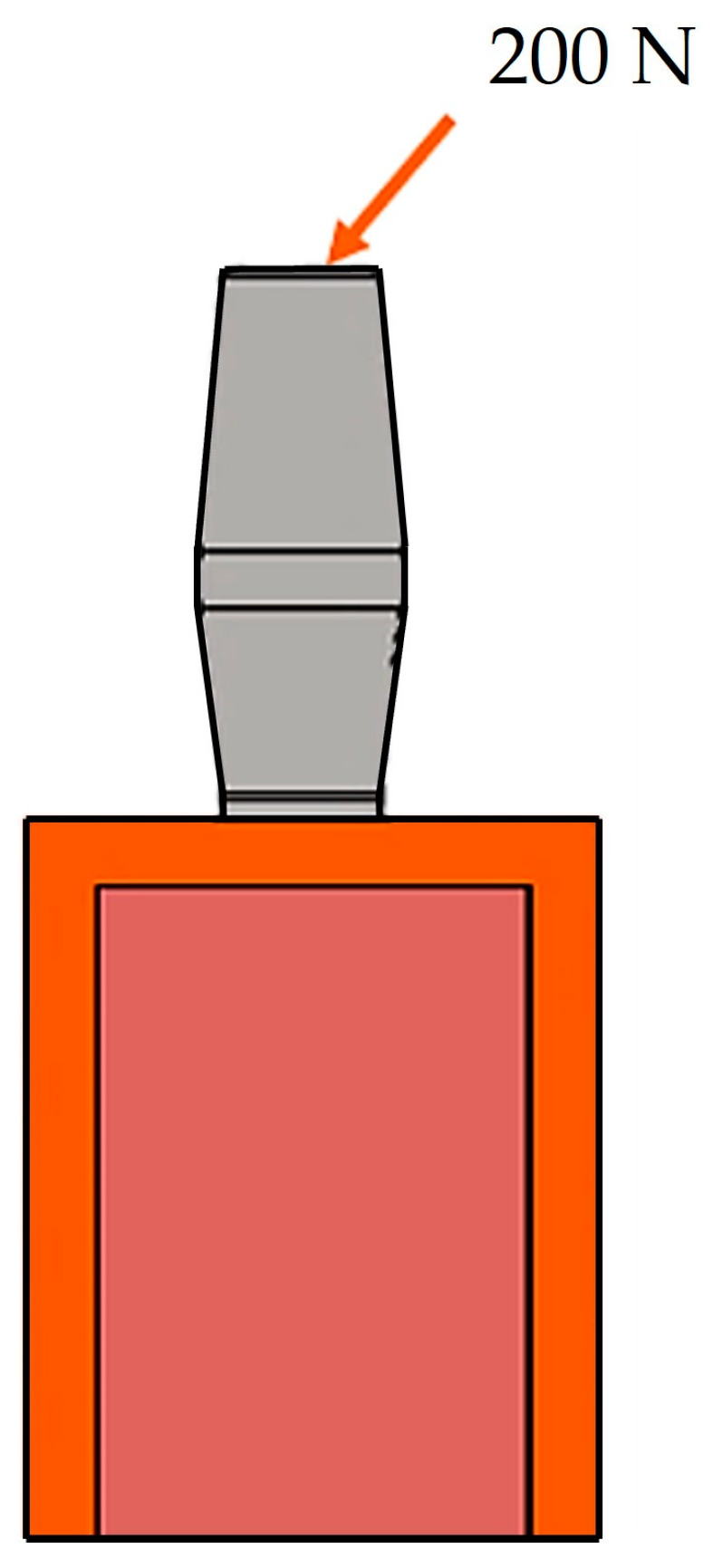
Application of an oblique load (200 N) on the abutment.

The loading conditions are shown in Figure 5, where a force of 200 N was applied at a 45° inclination with respect to the implant’s long axis [27]. The inferior surface of the model and the medial and distal planes of the bone were fully constrained.

In this study, the stress induced by applied forces on both the implant surface and the bone has been analyzed. The Von Mises criterion (based on equivalent stress) was employed to analyze the system’s stress, being particularly useful in situations involving multi-directional and time-varying loads. It also considered shear and normal stresses, providing a more accurate criterion than other models in the presence of complex loads [28]. Specifically, data concerning mathematical solutions have been converted into visual results as color gradients shifting from red to blue. The blue color indicates the minimum stress, whereas the red one the maximum stress. All the shades present in this range were considered as the stress variation. Subsequently, stress values were measured at different points collected from the studied models and then compared. Von Mises equivalent stress levels were used to identify points with the greatest stress for the model analyzed [29]. This investigative approach allowed the comprehension of the biomechanical behavior of the bone–implant system.

Then, after determining the stress acting on the bone, it could be compared with the stress values proposed by Frost [6] to assess the likelihood of bone resorption.

## 3. Results

In this study, the Von Mises strength criterion was applied using the yield strength for titanium (860 MPa) [21] and the tensile strength for bone (40 MPa) [23] as limit stresses.

Stress values were collected and compared at various points in the three models, and locations with the highest stress were determined based on Von Mises equivalent stress levels. The obtained results provided valuable insights into the areas where stress concentrations occur within the implant and surrounding tissues.

**Figure 6:**
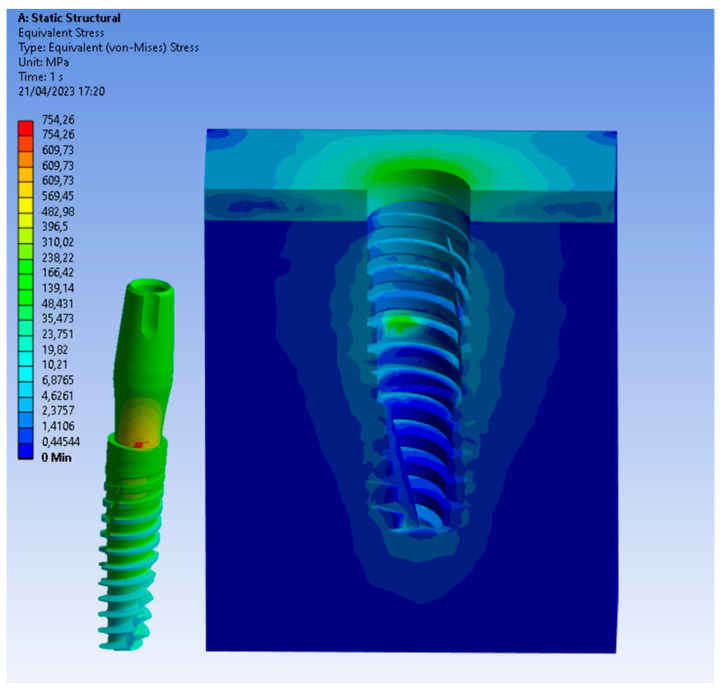
Von Mises stress at the equicrestal level (0 mm).

**Figure 7:**
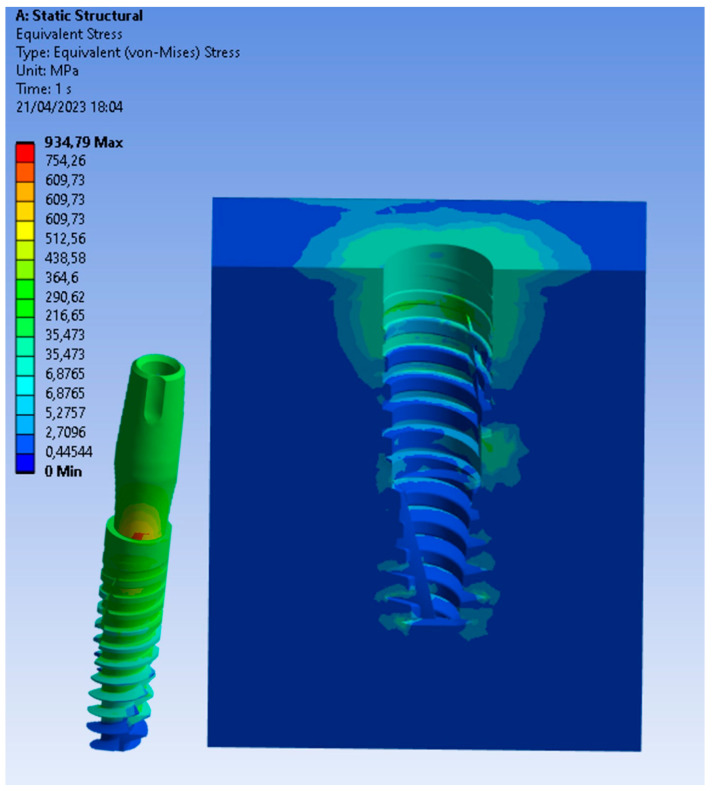
Von Mises stress at the subcrestal level (−1 mm).

**Figure 8:**
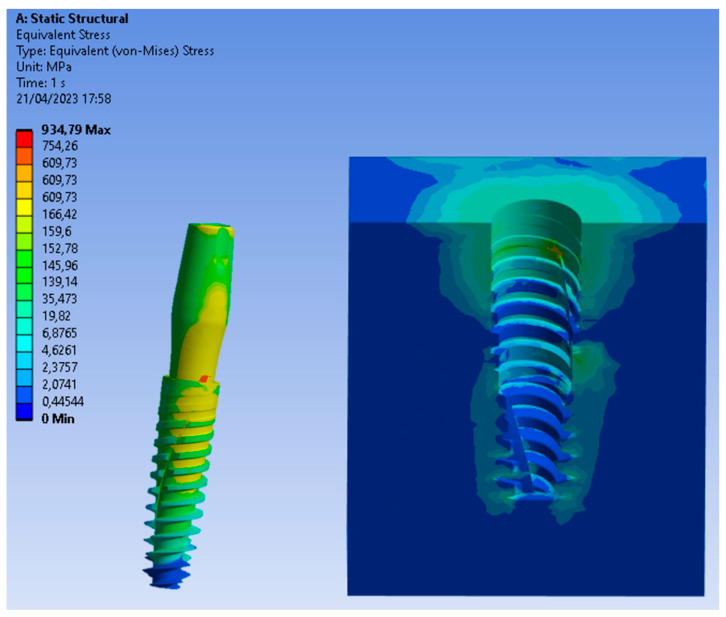
Von Mises stress at the subcrestal level (−2 mm).

Particularly, Figure 6, Figure 7 and Figure 8 present a visual representation of the Von Mises stress distribution. These stress maps provide a quick visualization of areas that exhibit higher stress levels, enabling a clearer understanding of load characteristics and potential areas that may lead to mechanical complications.

In the model with the implant placed equicrestally, the cortical bone exhibited a higher stress value of 40 MPa, as shown in Figure 6. On the other hand, placing the implant in a subcrestal position had a notable impact on stress within the peri-implant tissue. Indeed, in this position, the stress distribution was more homogeneous and lower than in the equicrestal placement, as shown in Figure 7 and Figure 8. This result suggested that subcrestal implant placement by approximately 1.5 mm contributes to the creation of a favorable biomechanical environment around the implant, potentially reducing the risk of complications such as bone resorption, thereby promoting better long-term implant stability.

In Figure 8, showing the implant positioned 2 mm subcrestally, an increase in stress was evident in the apical area of the bone. Comparatively, in an equicrestal placement where approximately 10 MPa values were observed in the apical area of the bone, a subcrestal positioning of 2 mm resulted in stress values around 30 MPa. This indicated that the increased stress in the apical area of the implant may promote bone regrowth, but concurrently, a significant decrease in stress (14 MPa) occurred in the cortical area compared to the 40 MPa recorded with an equicrestal implant placement. Considering that bone resorption commonly occurs in the crestal area of the implant due to overload or stress, it suggested that an equicrestal positioning of the implant may more readily lead to crestal bone resorption. Furthermore, it should be considered that according to Frost’s theory [6], a stress of about 46 MPa on a D2 bone type is considered a critical limit to mitigate the risk of fractures and issues such as overload-induced resorption.

**Figure 9:**
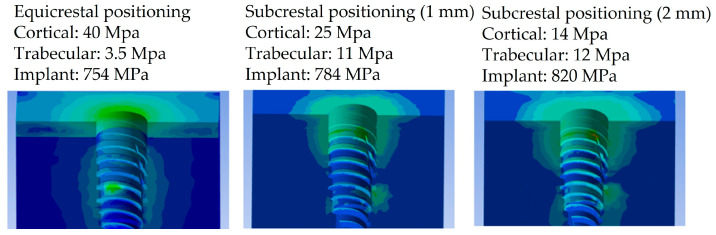
Summary of Von Mises stress results.

Upon closer examination of Figure 9, it was evident that stress distribution on the implant was more uniform with equicrestal implant positioning. In contrast, subcrestal placements at 1 mm and 2 mm exhibited tension concentrations in the intermediate zone between the crestal and subcrestal sections of the implant. This uneven stress distribution may not stimulate the cortical bone and trabecular bone uniformly, resulting in localized areas of stress overload. The application of an oblique loading subjected the system to stresses not only along its axis of symmetry but also in a transverse direction. This condition increased flexion in the connection zone between the abutment and the implant, leading to a concentration of tension on the lower area of the abutment. As can be seen from Figure 6, Figure 7 and Figure 8, this stress approached the yield stress of the titanium alloy Ti6Al4V. In general, the application of an oblique load on the implant reveals higher Von Mises stresses.

Additionally, it was noticeable on the implant that, for the same applied force, subcrestal positioning induced an increase in stress. For instance, in Figure 9, with equicrestal positioning, the maximum stress on the implant was 754 MPa, while increasing the insertion depth it resulted in a stress increase up to 820 MPa.

It can be asserted that positioning at a depth of 2 mm represents the most unfavorable condition, leading to alterations in stress on the implant and a decrease in stress in the cortical bone area.

## 4. Discussion

The question of the most appropriate implant positioning, whether more coronally or apically relative to the alveolar crest, remains highly controversial in the literature, with numerous studies presenting conflicting outcomes. For instance, Degidi et al. [16] suggested that in cases of tapered single implants placed in a subcrestal position and restored with an immediate prosthesis, the use of a non-removable abutment improved peri-implant stability in both soft and hard tissues. This approach is commonly referred to as the “one-abutment-one-time” technique. On the contrary, other researchers indicated that implants placed at the crestal level exhibited greater and improved stability of the peri-implant bone [30].

However, contrasting findings have been reported, with no statistically significant difference observed in marginal bone loss when comparing crestally and subcrestally placed implants [31,32]. These results were corroborated by a clinical study involving 62 implants inserted in 27 patients, where no significant differences in marginal bone loss between equicrestal and subcrestal implants were found [33]. In contrast, another clinical study reported that implants in a subcrestal position tended to maintain and conserve crestal peri-implant bone for longer periods compared to equicrestal placements [34]. This observation was further supported by a reduced probability of implant thread exposure in subcrestally located implants [35]. Contrarily, in the literature, a higher amount of marginal bone loss with implants inserted in a 2 mm subcrestal position was also reported [36].

Furthermore, animal experimental studies have demonstrated several positive outcomes associated with subcrestal implant positioning. These include beneficial effects on the remodeling of peri-implant crestal bone in Morse cone connection implants [18], the absence of consistently negative effects on the peri-implant bone with the sinking of two-piece implants [37], a smaller degree of marginal bone loss around 1 mm subcrestal implants [38], significantly reduced peri-implant bone loss with 1.5 mm and 3 mm subcrestally placed implants compared to equicrestal implants [39], and the effectiveness of preserving and maintaining inter-implant crestal bone by placing adjacent Morse cone implants with platform switching in a 1.5 mm subcrestal position [40]. Moreover, histological examination of animal experiments revealed that in subcrestally positioned Morse cone implants, the bone was overgrowing the micro-gap and touching the abutment surface [14].

Similar results were reported in human-retrieved Morse cone implants. In fact, pre-existent and newly deposited bone were found overgrowing the implant shoulder and the implant–abutment junction in all implants positioned subcrestally, while in equicrestal implants, a 0.5–1.5 mm of resorption was found. For instance, in an immediately loaded implant retrieved after a 1-month loading period, newly deposited bone was present at 2 mm over the implant shoulder, and the pre-existent bone had not undergone resorption [16].

Even systematic reviews of the literature with meta-analyses have been unable to provide a clear and definitive answer. It may be assumed that a lower amount of bone loss was found in equicrestally situated implants, but only before the abutment connection, whereas subcrestal implants showed the lowest percentage of bone resorption after the abutment connection [9]. For instance, in a systematic review and meta-analysis of 16 studies, including both randomized control trial (RCT) and not-RCT, Palacios et al. [41] found no differences in 10 studies, higher bone resorption in a subcrestal position in 3 studies, and lower bone resorption in implants placed subcrestally in another 3 studies. They concluded that no differences in marginal bone resorption were found in most of the reviewed studies. On the contrary, Valles et al. [9], in a systematic review with meta-analysis of 7 human and 7 animal studies, reported a lesser quantity of bone resorption in subcrestally situated implants. In general, whether placing an implant above or below the alveolar crest, there was an increase in stresses on the cortical bone [41]. Stresses on the peri-implant cortical bone tended to decrease with the increasing depth of implant positioning. Consequently, in subcrestal implants, the highest bone stresses were located away from the cortical region, with the most significant reduction in stresses occurring at depths ranging from 0.6 to 1.2 mm [42].

FEA simulations are commonly employed in biomechanical applications to understand the clinical factors that may contribute to the success or failure of an implant. However, it is essential to recognize that FEA studies are numerical analyses, providing approximations of the actual component behavior rather than a true representation. The results obtained through FEA should be carefully assessed by comparing them with clinical outcomes. This approach, for instance, can be utilized to evaluate stress and deformation within the bone, offering advantages over the use of strain gauges that only detect alterations in surface deformation. The acquired data can aid designers in optimizing the implant and offer valuable insights to clinicians regarding insertion techniques. Stress and strain are crucial parameters for crestal bone maintenance and implant survival, and studying these stresses directly on the patient is impractical. Therefore, the FEA method becomes useful in evaluating these parameters on the implant [43]. Despite the advantages, conflicting results have also emerged in FEA studies. For instance, Li et al. [44], when evaluating implants inserted equicrestally and at 0.5 and 1 mm below the alveolar crest, reported fewer strains in the bone around equicrestal and −0.5 mm placed implants than at −1 mm placed implants. Similarly, in an FEA study focusing on D4 bone type [45], the lowest amount of stress was found in 0.5 mm subcrestally placed implants. On the contrary, Macedo et al. [20] observed increased stresses in subcrestal implants, while a better distribution of stresses around crestal implants was reported.

The present study, utilizing the Finite Element method, assessed the stress distribution on bone and implants positioned at crestal and subcrestal depths of 1 mm and 2 mm under a 200 N load inclined at 45° to the apical direction of the implant. The results indicated that both the load inclination and implant placement influenced the mechanical behavior of the bone–implant structure. Consequently, the null hypothesis was rejected.

Understanding how masticatory forces are transmitted to the prosthetic components and, ultimately, to the surrounding bone is crucial for long-term implant success. Stress distribution is influenced by various clinical and mechanical factors, including the type of load applied (axial or inclined), implant shape, and material. For instance, when using a material with a stiffness similar to bone, the load is primarily distributed at the bone–implant interface, leading to increased stress on the bone in contact with the implant. In addition, the bone type plays a role in stress distribution, with denser bones (e.g., D1 type) absorbing more stress than less dense bones (e.g., D3 type) [46]. In D3 bones, stress tends to concentrate in the apical area of the implant, while in D1 bones, the stress is focused in the cortical area. This study considered a standard case (D2 bone) according to the Misch classification [4]. Moreover, the depth of implant insertion in relation to the bone level is increasingly becoming crucial for clinicians. It directly influences the preservation of both soft and hard tissues and also has a significant impact on the aesthetic results achievable through bracketing. All these factors impact implant stability and the bone’s ability to remodel around the implant surface [46].

The findings of this study indicated an increase in stress within the cortical bone when the implant was placed equicrestally. To achieve favorable results in terms of bone stress and ensure long-term durability, a positioning at −1 mm can be considered optimal. Subcrestal positioning led to increased stress in the apical region, potentially triggering a bone-preserving mechanism that promotes stability. However, if this stress on a D2 bone type exceeds approximately 46 MPa, bone absorption due to overload can occur [6]. These results aligned with other studies indicating that subcrestal positioning offers a favorable biomechanical environment. Moreover, subcrestal positioning ensures better bone preservation and enhances the stability of soft tissues around the implant.

In any case, future studies should further explore the influence of bone quality on stress transmission with various implant locations, such as changes in density or cortical bone thickness.

## 5. Conclusions

This study thoroughly examined the impact of implant placement depth on stress distribution within the cortical and cancellous peri-implant bone. Specifically, it investigated the effects of positioning Morse cone dental implants at equicrestal and subcrestal levels, with depths of −1 mm and −2 mm, using advanced 3D FEA techniques.

Within the limitations of a FEA study, the results revealed that the maximum Von Mises stresses were observed within the cortical bone around the equicrestally positioned implant, followed by the 1 mm subcrestally placed implant, and then by the 2 mm subcrestally inserted implant. When comparing cortical and cancellous bones, the maximum stresses were found within the cortical bone.

Subcrestal placement of a Morse cone implant–abutment connection (ranging between −1 and −2 mm) could be recommended as an approach to reduce the peri-implant bone resorption and achieve longer-term implant success.

## Figures and Tables

**Table 1:** Material properties used in this Finite Element Analysis (FEA) study.

Material	Young’s Modulus (GPa)	Poisson’s Ratio
Cortical bone	13.70	0.30
Cancellous bone	1.37	0.30
Titanium	117.00	0.30

## Data Availability

All experimental data to support the findings of this study are available from the corresponding authors upon request.

## References

[1] Duyck J., Naert I., Van Oosterwyck H., Van der Sloten J., De Cooman M., Lievens S., Puers B. (1997). Biomechanics of oral implants: A review of the literature. Technol. Health Care.

[2] Duyck J., Naert I., Rønold H.J., Ellingsen J.E., Van Oosterwyck H., Sloten J.V. (2001). The influence of static and dynamic loading on marginal bone reactions around osseointegrated implants: An animal experimental study. Clin. Oral Implant. Res..

[3] Tarnow D., Cho S., Wallace S. (2000). The effect of inter-implant distance on the height of inter-implant bone crest. J. Periodontol..

[4] Misch C.E., Suzuki J.B., Misch-Dietsh F.M., Bidez M.W. (2005). A Positive correlation between occlusal trauma and peri-implant bone loss: Literature support. Implant. Dent..

[5] Qian L., Todo M., Matsushita Y., Koyano K. (2009). Finite element analysis of bone resorption around dental implant. J. Biomech. Sci. Eng..

[6] Frost H.M. (1992). Perspectives: Bone’s mechanical usage windows. Bone Miner..

[7] Qian L., Todo M., Matsushita Y., Koyano K. (2009). Effects of implant diameter, insertion depth and loading angle on stress/strain fields in implant/jawbone systems. Int. J. Oral Maxillofac. Implant..

[8] Cruz R.S., Lemos C.A.A., de Luna Gomes J.M., Fernandes E., Oliveira H.F., Pellizzer E.P., Verri F.R. (2022). (Clinical comparison between crestal and subcrestal dental implants: A systematic review and meta-analysis. J. Prosthet. Dent..

[9] Valles C., Rodríguez-Ciurana X., Clementini M., Baglivo M., Paniagua B., Nart J. (2018). Influence of subcrestal implant placement compared with equicrestal position on the peri-implant hard and soft tissues around platform-switched implants: A systematic review and meta-analysis. Clin. Oral Investig..

[10] D’Ercole S., Tripodi D., Marzo G., Bernardi S., Continenza M.A., Piattelli A., Iaculli F., Mummolo S. (2015). Microleakage of bacteria in different implant-abutment assemblies: An in vitro study. J. Appl. Biomater. Funct. Mater..

[11] Weng D., Nagata M.J.H., Bell M., Bosco A.F., De Melo L.G.N., Richter E. (2008). Influence of microgap location and configuration on the periimplant bone morphology in submerged implants. An experimental study in dogs. Clin. Oral Implant. Res..

[12] Weng D., Nagata M.J., Leite C.M., de Melo L.G., Bosco A.F. (2011). Influence of microgap location and configuration on radiographic bone loss in nonsubmerged implants: An experimental study in dogs. Int. J. Prosthodont..

[13] Weng D., Nagata M.J., Bosco A.F., de Melo L.G. (2011). Influence of microgap location and configuration on radiographic bone loss around submerged implants: An experimental study in dogs. Int. J. Oral. Maxillofac. Implants.

[14] Weng D., Nagata M.J., Bell M., de Melo L.G., Bosco A.F. (2010). Influence of microgap location and configuration on peri-implant bone morphology in nonsubmerged implants: An experimental study in dogs. Int. J. Oral. Maxillofac. Implants.

[15] Degidi M., Nardi D., Daprile G., Piattelli A. (2014). Nonremoval of immediate abutments in cases involving subcrestally placed postextractive tapered single implants: A randomized controlled clinical study. Clin. Implant. Dent. Relat. Res..

[16] Degidi M., Perrotti V., Shibli J.A., Novaes A.B., Piattelli A., Iezzi G. (2011). Equicrestal and subcrestal dental implants: A histologic and histomorphometric evaluation of nine retrieved human implants. J. Periodontol..

[17] Fetner M., Fetner A., Koutouzis T., Clozza E., Tovar N., Sarendranath A., Coelho P., Neiva K., Janal M., Neiva R. (2015). The Effects of Subcrestal Implant Placement on Crestal Bone Levels and Bone-to-Abutment Contact: A Microcomputed Tomographic and Histologic Study in Dogs. Int. J. Oral Maxillofac. Implant..

[18] de Castro D.S.M., de Araujo M.A.R., Benfatti C.A.M., Araujo C.d.R.P.d., Piattelli A., Perrotti V., Iezzi G. (2014). Comparative histological and histomorphometrical evaluation of marginal bone resorption around external hexagon and Morse cone implants: An experimental study in dogs. Implant. Dent..

[19] Santonocito D., Nicita F., Risitano G. (2021). A Parametric Study on a Dental Implant Geometry Influence on Bone Remodelling through a Numerical Algorithm. Prosthesis.

[20] Macedo J.P., Pereira J., Faria J., Souza J.C.M., Alves J.L., López-López J., Henriques B. (2018). Finite element analysis of peri-implant bone volume affected by stresses around Morse taper implants: Effects of implant positioning to the bone crest. Comput. Methods Biomech. Biomed. Eng..

[21] Callea C., Ceddia M., Piattelli A., Specchiulli A., Trentadue B. (2023). Finite Element Analysis (FEA) for a Different Type of Cono-in Dental Implant. Appl. Sci..

[22] Di Pietro N., Ceddia M., Romasco T., De Bortoli Junior N., Mello B.F., Tumedei M., Specchiulli A., Piattelli A., Trentadue B. (2023). Finite Element Analysis (FEA) of the Stress and Strain Distribution in Cone-Morse Implant–Abutment Connection Implants Placed Equicrestally and Subcrestally. Appl. Sci..

[23] Cipollina A., Ceddia M., Di Pietro N., Inchingolo F., Tumedei M., Romasco T., Piattelli A., Specchiulli A., Trentadue B. (2023). Finite Element Analysis (FEA) of a Premaxillary Device: A New Type of Subperiosteal Implant to Treat Severe Atrophy of the Maxilla. Biomimetics.

[24] Kheiralla L.S., Younis J.F. (2014). Peri-implant biomechanical responses to standard, short-wide, and mini implants supporting single crowns under axial and off-axial loading (an in vitro study). J. Oral Implant..

[25] Kang N., Wu Y.-Y., Gong P., Yue L., Ou G.-M. (2014). A study of force distribution of loading stresses on implant–bone interface on short implant length using 3-dimensional finite element analysis. Oral Surg. Oral Med. Oral Pathol. Oral Radiol..

[26] Toniollo M.B., Macedo A.P., Rodrigues R.C.S., Ribeiro R.F., Mattos M.d.G.C.d. (2013). A three-dimensional finite element analysis of the stress distribution on morse taper implants surface. J. Prosthodont. Res..

[27] Gatti C., Gatti F., Silvestri M., Mintrone F., Rossi R., Tridondani G., Piacentini G., Borrelli P. (2018). A Prospective Multicenter Study on Radiographic Crestal Bone Changes Around Dental Implants Placed at Crestal or Subcrestal Level: One-Year Findings. Int. J. Oral Maxillofac. Implant..

[28] Fiorillo L., Cicciù M., D’amico C., Mauceri R., Oteri G., Cervino G. (2020). Finite Element Method and Von Mises Investigation on Bone Response to Dynamic Stress with a Novel Conical Dental Implant Connection. BioMed Res. Int..

[29] Baggi L., Di Girolamo M., Vairo G., Sannino G. (2013). Comparative evaluation of osseointegrated dental implants based on platform-switching concept: Influence of diameter, length, thread shape, and in-bone positioning depth on stress-based perfor-mance. Comput. Math. Methods Med..

[30] Sargolzaie N., Zarch H.H., Arab H., Koohestani T., Ramandi M.F. (2022). Marginal bone loss around crestal or subcrestal dental implants: Prospective clinical study. J. Korean Assoc. Oral Maxillofac. Surg..

[31] Nagarajan B., Murthy V., Livingstone D., Surendra M.P., Jayaraman S. (2015). Evaluation of Crestal Bone Loss Around Implants Placed at Equicrestal and Subcrestal Levels Before Loading: A Prospective Clinical Study. J. Clin. Diagn Res..

[32] Chatterjee P., Shashikala R., Navneetham A. (2022). Comparative Study of the Crestal vs. Subcrestal Placement of Dental Implants via Radiographic and Clinical Evaluation. J. Contemp. Dent. Pract..

[33] Palacios-Garzón N., Mauri-Obradors E., Ayuso-Montero R., Velasco-Ortega E., Anglada-Cantarell J.M., López-López J. (2022). Marginal Bone Loss in Internal Conical Connection Implants Placed at the Crestal and Subcrestal Levels before Prosthetic Loading: A Randomized Clinical Study. Materials.

[34] Ercoli C., Jammal G., Buyers M., Tsigarida A.A., Chochlidakis K.M., Feng C., Caton J. (2017). Influence of Apico-Coronal Implant Placement on Post-Surgical Crestal Bone Loss in Humans. J. Periodontol..

[35] Jain S., Mattoo K., Khalid I., Baig F.A.H., Kota M.Z., Ishfaq M., Ibrahim M., Hassan S. (2023). A study of 42 partially edentulous patients with single-crown restorations and implants to compare bone loss between crestal and subcrestal endosseous implant placement. Med. Sci. Monit..

[36] Pontes A.E.F., Ribeiro F.S., Iezzi G., Pires J.R., Zuza E.P., Piattelli A., Junior E.M. (2014). Bone-implant contact around crestal and subcrestal dental implants submitted to immediate and conventional loading. Sci. World J..

[37] Todescan F.F., Pustiglioni F.E., Imbronito A.V., Albrektsson T., Gioso M. (2002). Influence of the microgap in the peri-implant hard and soft tissues: A histomorphometric study in dogs. Int. J. Oral. Maxillofac. Implants.

[38] Huang B., Meng H., Zhu W., Witek L., Tovar N., Coelho P.G. (2015). Influence of placement depth on bone remodeling around tapered internal connection implants: A histologic study in dogs. Clin. Oral Implant. Res..

[39] Barros R.R.M., Novaes A.B., Muglia V.A., Iezzi G., Piattelli A. (2010). Influence of interimplant distances and placement depth on peri-implant bone remodeling of adjacent and immediately loaded Morse cone connection implants: A histomorphometric study in dogs. Clin. Oral Implant. Res..

[40] Saleh M.H.A., Ravidà A., del Amo F.S., Lin G., Asa’Ad F., Wang H. (2018). The effect of implant-abutment junction position on crestal bone loss: A systematic review and meta-analysis. Clin. Implant. Dent. Relat. Res..

[41] Palacios-Garzón N., Velasco-Ortega E., López-López J. (2019). Bone Loss in Implants Placed at Subcrestal and Crestal Level: A Systematic Review and Meta-Analysis. Materials.

[42] Chu C.-M., Huang H.-L., Hsu J.-T., Fuh L.-J. (2012). Influences of internal tapered abutment designs on bone stresses around a dental implant: Three-dimensional finite element method with statistical evaluation. J. Periodontol..

[43] de Carvalho N.A., de Almeida E.O., Rocha E.P., Freitas A.C., Anchieta R.B., Kina S. (2012). Short implant to support maxillary restorations: Bone stress analysis using regular and switching platform. J. Craniofac. Surg..

[44] Li R., Wu Z., Chen S., Li X., Wan Q., Xie G., Pei X. (2023). Biomechanical behavior analysis of four types of short implants with different placement depths using the finite element method. J. Prosthet. Dent..

[45] Sesha M.R., Sunduram R., Abdelmagyd H.A.E. (2020). Biomechanical Evaluation of Stress Distribution in Subcrestal Placed Platform-Switched Short Dental Implants in D4 Bone: In Vitro Finite-Element Model Study. J. Pharm. Bioallied Sci..

[46] Körtvélyessy G., Szabó L., Pelsőczi-Kovács I., Tarjányi T., Tóth Z., Kárpáti K., Matusovits D., Hangyási B.D., Baráth Z. (2023). Different Conical Angle Connection of Implant and Abutment Behavior: A Static and Dynamic Load Test and Finite Element Analysis Study. Materials.

